# Gradual consolidation of skilled sequential movements in primary motor cortex of non-human primates

**DOI:** 10.1101/2024.10.06.616850

**Published:** 2026-01-21

**Authors:** Machiko Ohbayashi, Nathalie Picard

**Affiliations:** 1. Department of Neurobiology, University of Pittsburgh School of Medicine, Pittsburgh, Pennsylvania 15261, USA.; 2. Systems Neuroscience Center, University of Pittsburgh, Pittsburgh, Pennsylvania 15261, USA.

## Abstract

Many of our daily actions rely on skilled sequential movements. Sequence performance improves with practice and can reach expert levels with years of repeated practice, truly exemplifying the saying “Practice makes perfect”. Human and non-human primate studies have shown structural and functional changes in the primary motor cortex (M1) following extended practice of sequential movements, suggesting M1’s involvement in acquisition and retention of skilled sequences. However, it has been challenging to causally examine M1’s role in sequence learning, as inactivation or lesion of M1 impairs movement production. Here, we causally examined M1’s contribution to sequence learning by locally inhibiting protein synthesis in M1. Our results show that protein synthesis inhibition in M1 disrupted memory-guided sequential movements at all stages of learning without affecting movement production, though the effects decreased with continued practice. These findings suggest that neural traces for sequential movements are repeatedly consolidated in M1 through protein synthesis, with the rate of consolidation slowing as learning progresses.

## Teaser

Repetitive practice strengthens neural representations of sequential movements in primary motor cortex of monkeys.

## Introduction

Many daily activities, such as playing a musical instrument and typing, rely on attaining a high level of proficiency in performing sequential movements. The sequential movements can be acquired and improved to an expert level through extensive practice, eventually becoming a stable motor skill. Once the structure of a sequence is learned during the early phase of learning, continued daily practice over an extended period leads to gradual improvement in speed and accuracy, key indicators of motor skill learning. This slow incremental learning is considered a fundamental aspect of motor skill development, capturing the idea that “practice makes perfect.”

Emerging evidence indicates that multiple cortical motor areas alter their activity during the learning of sequential movements (1–11). The primary motor cortex (M1), in particular, exhibits functional and structural changes associated with the learning of the sequential movements, not only during the early stages of learning but also after extensive practice. In humans, short-term training induces measurable changes in fMRI activity within M1 (1–9, 12, 13). Similarly, studies in monkeys demonstrate that neural activity in M1 is modulated by sequence components during the early stage of learning (14, 15).

With extensive practice, additional changes in M1 have been reported in both human and non-human primate. In humans, years of extensive practice are associated with structural modifications and altered functional activation of M1 (9, 11, 16–20). For instance, professional musicians who have spent years practicing complex sequential movements exhibit reduced or more focused M1 activation during the performance of sequential tasks compared with non-musicians or amateurs (16–20). This reduced activation is considered as evidence of increased efficiency of neural circuits, or the need for a smaller number of active neurons to perform a highly trained set of sequential movements. Furthermore, the volume of M1 has been reported to be larger in professional musicians compared to amateurs or non-musicians (21–28). These structural changes have been proposed to arise form synaptic processes, including intracortical remodeling of dendritic spines and axonal terminals, glial hypertrophy, and synaptogenesis as a result of extensive practice (24–26, 29). Similarly, plasticity of the white matter structure of M1 has been correlated with the amount of time devoted to practice, suggesting that myelination may be increased by neural activity in fiber tracts during training (30, 31).

In non-human primates, after years of extensive training, M1 neurons were differentially active during the performance of visually guided and internally generated sequential reaching (32, 33). Furthermore, the uptake of 2DG, a measure associated with presynaptic activity, was lower in M1 of monkeys that performed highly practiced, internally generated sequences of movements compared with M1 of monkeys that performed visually guided reaching (33). Changes in M1 observed at the levels of neuronal activity, functional activation, and gray matter structure in humans and non-human primates may all reflect plasticity underlying the learning of sequential movements through extended, repetitive practice.

Together, these diverse findings converge to the concept that M1 contributes to the learning and maintenance of motor skills such as sequential movements through extensive practice. While these studies demonstrate that activity patterns in M1 change both during the early phase of learning and after extensive practice, they do not conclusively indicate whether M1 is critical for sequence learning nor when and how plasticity occurs within M1 during learning. The change after extensive training could be the outcome of plasticity during the early phase of the training. Thus, it remains unclear whether M1 undergoes changes throughout all phases of learning or only during specific stages, and whether M1 plays a critical role in learning of sequential movements. Moreover, the mechanisms by which M1 contributes to the slow, gradual improvements in sequence performance are not yet fully understood.

A traditional approach to these questions would be to assess the effect of lesion or inactivation of M1 on learning performance. However, M1 poses a special challenge to causally examine when and how plastic changes occur during learning, because M1 is critical for implementing motor output. A lesion or inactivation of M1 will abolish the motor commands to the spinal cord that generate muscle activity, so that it would be impossible to evaluate M1’s involvement in learning. Therefore, we targeted information storage in M1 instead of motor output. We injected an inhibitor for protein synthesis in M1 to interfere with information storage and, consequently, learning.

Inhibitors for protein synthesis have been widely used to dissect and analyze memory systems in rodents, such as fear conditioning. Local injection of an inhibitor of protein synthesis (e.g., anisomycin) into amygdala, hippocampus or motor cortex of rodents disrupted the learning, maintenance or reconsolidation of memory (34–44). These studies indicated that protein synthesis inhibition can interfere with formation and storage of memory. Building on this approach, we previously injected a protein synthesis inhibitor into M1 of monkeys after ~100 days of training and found that it successfully disrupted performance of well-trained sequential movements without affecting the performance of visually guided reaching (45). Here, we applied this approach to examine when and how M1 contributes to learning of sequential movements during repetitive practice over extensive time in non-human primates.

## Results

### Learning of sequential reaching task

**Fig. 1:**
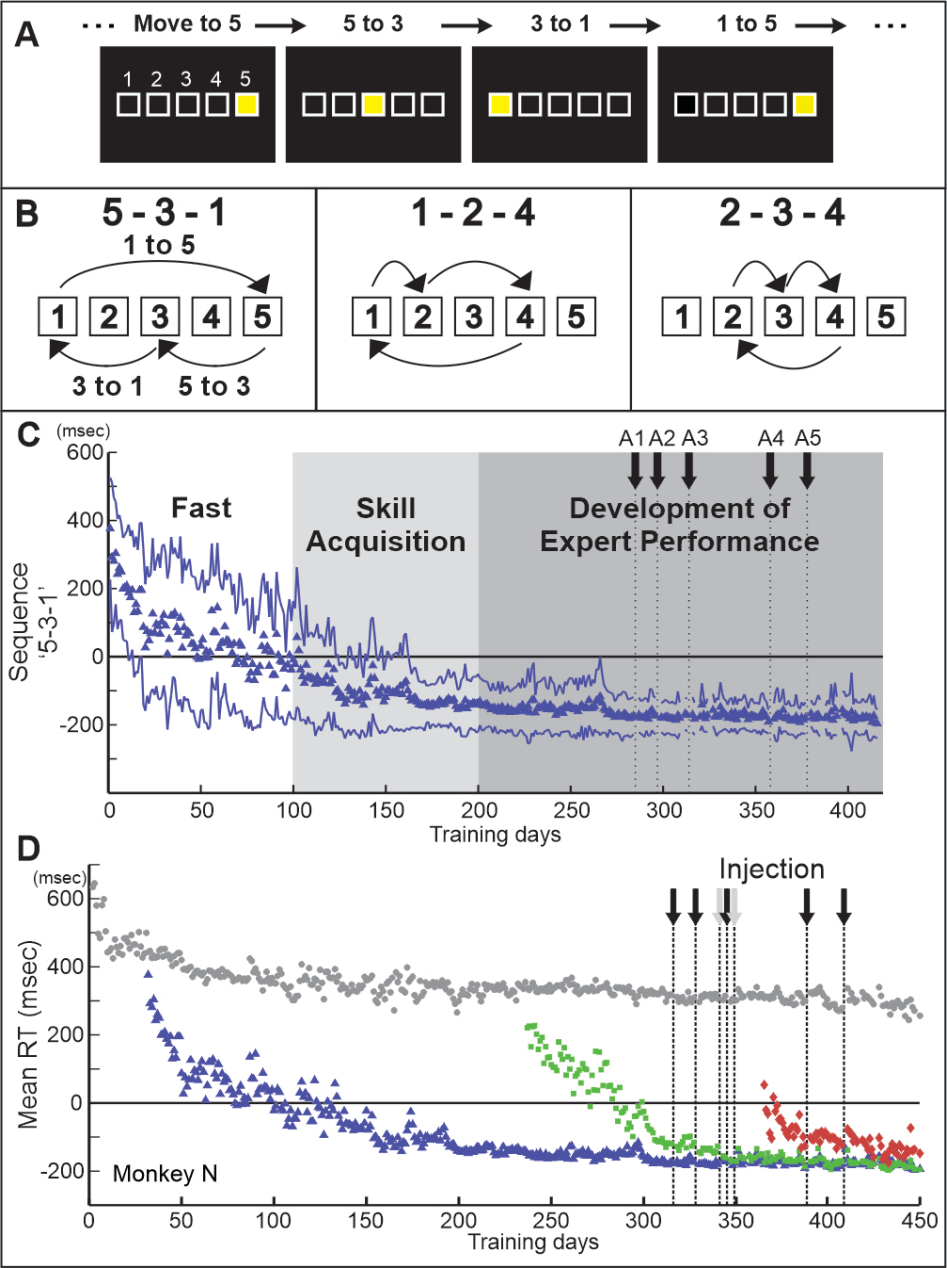
Sequential Reaching Task. A. Sequential reaching task. B. Repeating sequences in the Repeating task. C. Response Time (RT) of sequence ‘5–3-1’ of the Repeating task. We arbitrary divided the learning curve on the Repeating task into 3 periods based on the slope of RT changes. The first period corresponds to the ‘Fast Learning’ phase where a large RT reduction occurs in a short time frame. The second and third periods are defined as the early and late epochs of the Slow Learning phase and correspond to ‘Skill Acquisition’ and the ‘Development of Expert Performance’. D. The introductions of the new sequences were staggered at ~ 100–200 days intervals. This staggered training design enabled us to compare the effect of pharmacological injections between the ‘Long-term’ trained and ‘Short-term’ trained sequences in a single injection session. Gray circle: RT of the Random task; Blue triangle: RT of the Repeating sequence “5–3-1”; blue lines in C: mean +/− s.d.; Green square: RT of the Repeating sequence “1–2-4”; Red diamond: RT of the Repeating sequence “2–3-4’: Arrow: injection of anisomycin.

We trained three monkeys (*Cebus apella*) on two types of reaching tasks (Random and Repeating tasks, Fig. 1, for details see Materials and methods). In the Repeating task, we presented visual instruction cues according to a repeating sequence of 3 elements (e.g., 5–3-1–5-3–1- ..., Fig. 1A, B) 400 msec after monkey’s contact of the preceding target. If the monkey made a correct response during the 400 msec delay, the instruction cue was not presented, and the task proceeded to the next target in the sequence. With practice, monkeys memorized the sequence and performed the correct sequence in the Repeating task using predictive responses instead of using visual instructions to guide their reaching (Fig 1C, D). Thus, the monkeys performed memory-guided sequential movements during the Repeating task. As a control task, we trained the monkeys to perform the Radom task in which the visual instruction cues were presented in pseudo random order. During the Random task, cues were presented 100 ms after the monkey contacted the target, thereby preventing predictive responses. Thus, the monkeys performed reaching movements guided by visual cues (Random task). The two tasks were performed continuously in alternating blocks of 200–500 trials in each daily session. The monkeys predicted the targets in a Repeating sequence in more than 80% of trials after 50 days of training (Fig. 1C).

We trained monkeys to perform two to three sequences of the Repeating task. We added new sequences one at a time on a staggered schedule. A new sequence was introduced only after the previous one had been practiced for more than 50 days (Fig. 1D; see Methods). During each training session, the monkey performed each sequence and the Random task in alternating blocks of 200–500 trials. As a result, the training duration for each sequence differed at the time of experiments. For example, in Fig. 1D, on day 393, training duration for the first sequence was 357 days, 151 days for the second sequence, and 42 days for the third sequence. As shown in Figure 1D, Response Time (RT) for each sequence decreased independently as training proceeded, which provided time points when the sequences were in different learning phases. RT for the Random task (gray dots in Fig. 1D) reached a plateau after approximately 20 days of training. This staggered design enabled us to examine whether the effect of pharmacological injection depended on training duration and learning phase, both by comparing the effect on sequences within a single injection session in addition to a comparison across different injection sessions.

### Protein synthesis inhibition disrupted performance of memory guided sequences at all stages of learning, while sparing visually-guided reaching.

**Fig. 2:**
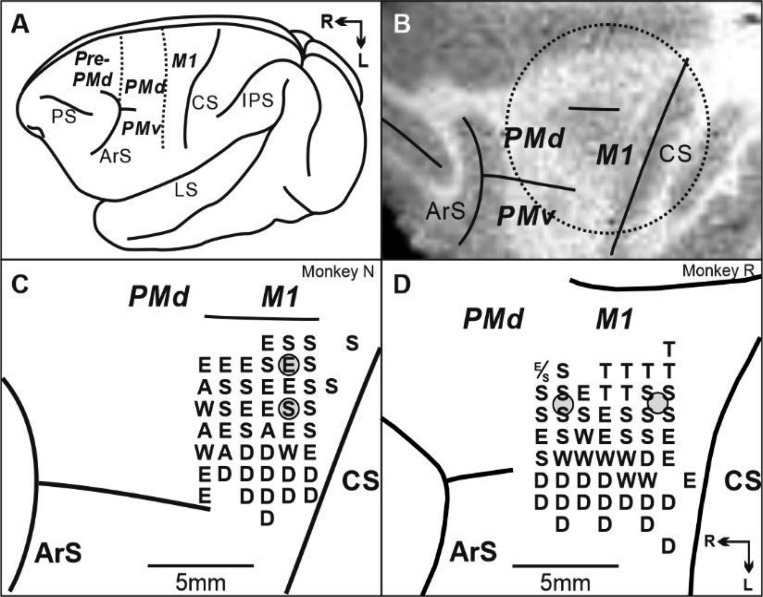
M1 location and the microstimulation maps of M1. A. Lateral view of cebus brain. Dashed lines indicate the M1-PMd border and the pre-PMd-PMd border. PS: principal sulcus; ArS: arcuate sulcus; CS: central sulcus; IPS: intra parietal sulcus; LS: lateral sulcus; pre-PMd: pre-dorsal premotor cortex; PMd: dorsal premotor cortex; M1: primary motor cortex. R: rostral; L: lateral. B. MRI image after the chamber implantation for monkey N. The dotted circle indicates the chamber outline. C, D. Intracortical stimulation map from monkey N and monkey R. Letters indicate the movements evoked at each site. S: Shoulder; E: Elbow; W: Wrist; D: Digit; Gray dots: representative injection sites.

When the monkey was able to perform the Random task and more than two sequences of the Repeating task, we made micro injections of the protein synthesis inhibitor anisomycin (100 mg/ml) at sites in M1 where intracortical microstimulation evoked shoulder or elbow movements (Fig. 2, see Methods). For each session, we analyzed the monkeys’ behavior before and after the injection, separately evaluating each movement in both the Random and Repeating task.

**Fig. 3:**
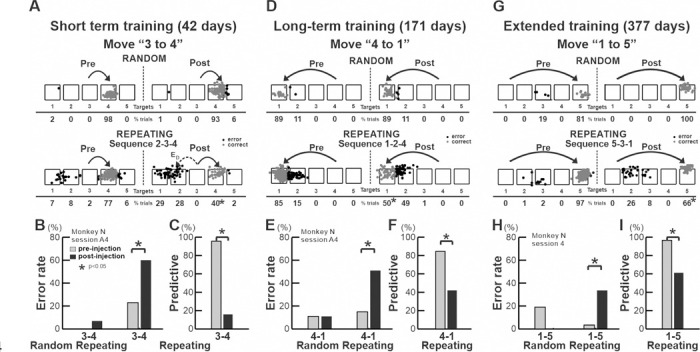
Effect of anisomycin injection on performance of the Random and Repeating tasks. A–C. Effect on movements “3 to 4” in the sequence “2–3-4” of the Repeating task after 42 days of training. A. Reaching end points during Random and Repeating tasks (pre- and post-injection). Gray dots: correct responses; black dots: error responses. E_D_: direction errors. Percentages of reaches ending at each target are shown below the targets. Touches between targets were assigned to the closest target. B. Errors increased only in the Repeating task after injection (Random: pre 96.96%, post 92.63%; *χ*^*2*^ *test*, *p*=0.09; Repeating: pre 77.24%, post 40.20%; *χ*^*2*^ *test*, *p* < 0.0001). C. Predictive responses decreased significantly after injection (pre 76.55%, post 12.74%; *χ*^*2*^ *test*, *p* < 0.0001). D–F. Effect on movement “4 to 1” in the sequence “1–2-4” of the Repeating task after 171 days of training, and corresponding movements in the Random task. D. Reaching end points for movements “4–1” during Random and Repeating tasks (pre- and post-injection). Gray dots: correct responses; black dots: error responses. E_A_: accuracy errors. E. Error rate significantly increased only in the Repeating task (Random: pre 89.47%, post 88.57%; *χ*^*2*^ *test*, *p* = 0.92; Repeating: pre 84.54%, post 50.38%; *χ*^*2*^ *test*, *p* < 0.0001). F. Predictive responses significantly decreased after injection (pre 84.54%, post 42.11%; *χ*^*2*^ *test, p* < 0.0001). G–I. Effect on movement “1 to 5” in the sequence “5–3-1” of the Repeating task after 377 days of training, and corresponding movements in the Random task. G. Reaching end points for movements “1–5” during Random and Repeating tasks (pre- and post-injection). Gray: correct; black: error. E_A_: accuracy errors. H. Errors significantly increased only in the Repeating task after injection (Random: pre 80.85%, post 100%; *χ*^*2*^ *test, p* = 0.006; Repeating: pre 96.51%, post 66.33%; *χ*^*2*^ *test, p* < 0.001). I. Predictive responses significantly decreased after injection (pre 95.58%, post 88.65%; *χ*^*2*^ *test, p* = 0.006). Note: Asterisks indicate *p* < 0.05.

In the injection session shown in Figure 3, monkey N performed three sequences with different training durations: a short duration (42 days) for sequence ‘2–3-4’ (Fig. 3 A-C), a long duration (151 days) for sequence ‘1–2-4’ (Fig. 3 D-F) and an extensive duration (357 days) for sequence ‘5–3-1’ (Fig. 3 G-I). The injection of anisomycin into M1 significantly disrupted performance of all sequences of the Repeating task. The injections resulted in a significant increase in the number of incorrect responses (Fig. 3B, E, H; *χ*^*2*^ *test, p*<0.0001) and a significant decrease in the number of predictive responses, an indicator of sequence learning, (Figure 3C, F, I; *χ*^*2*^ *test, p* < 0.01) for all moves across all three sequences of the Repeating task. The effect of anisomycin injection was more pronounced for certain movements within the learned sequences (increase of error rate: sequence ‘2–3-4’ - move ‘4–2’: 19.25%, ‘2–3’: −5.78%, ‘3–4’: 37.05%; sequence ‘1–2-4’ - move ‘4–1’: 34.16%, ‘1–2’: 6.89%, ‘2–4’: 9.68%; sequence ‘5–3-1’ - move ‘3–1’: 0.94%, ‘5–3’: 14.11%, ‘1–5’: 30.48%)(45). In contrast, performance of visually guided movements during the Random task was not significantly disrupted or slightly improved (Fig. 3 A, D, G, *χ*^*2*^ test, p>0.05).

Analysis of reaching endpoints on the screen revealed that the monkey made two types of error responses during the Repeating task: errors of accuracy and errors in direction. An accuracy error is a reach performed in the correct direction, but to an endpoint outside of the correct target. As shown in Figure 3D (sequence 1–2-4), after the anisomycin injection, during the Repeating task, the animal moved his arm in the correct direction to the left from target “4”, but the reach fell short in 50% of trials (Fig. 3D, bottom row). This type of error response was more frequent for movements starting from the targets at the edges of the touch monitor, target 1 and 5. A direction error is a reach performed in the direction opposite to the correct target. For example, movement “3–4” requires a rightward movement from target “3” to “4” (Fig. 3A). After the anisomycin injection, during the Repeating task, the animal instead moved his arm to the left from target “3” and made direction errors on 57% of the trials (Fig 3A, bottom row). Direction errors were not possible for movements starting from target 1 and 5, as movements in the opposite direction from these edge targets would fall outside the touch monitor. The direction errors suggest deficits in selecting the appropriate movement component within a sequence and in transitioning from one movement to the next. Given the increase of direction errors and unaffected performance in the Random task, we attribute the observed deficits in the Repeating task to the injection’s effect on memory for sequential movements.

We used the percentage of predictive trials and the Response Time (RT) as additional indicators to assess the effect of injections on task performance (Fig. 3C, F, I). After the anisomycin injection, RT for the affected movements in the Repeating task increased. Trials with longer RT (RT > 150 msec) were categorized as non-predictive responses (see Methods). The percentage of predictive trials of affected movements significantly decreased for all three sequences of the Repeating task (Figure 3C, F, I; *χ*^*2*^ *test, p* < 0.01). During the Repeating task, there were times when the monkey paused in mid-reach and redirected the arm after the visual cue was presented to make a correct response. This resulted in longer RTs and non-predictive responses. This behavior suggests that the monkey relied on the visual cue information to make a correct response, which was possible because the injection did not affect the performance of visually-guided movements (i.e., Random task). Therefore, the increase of RT after the anisomycin injection further supports the conclusion that the injection disrupted the memory-guided sequential movements.

**Fig. 4:**
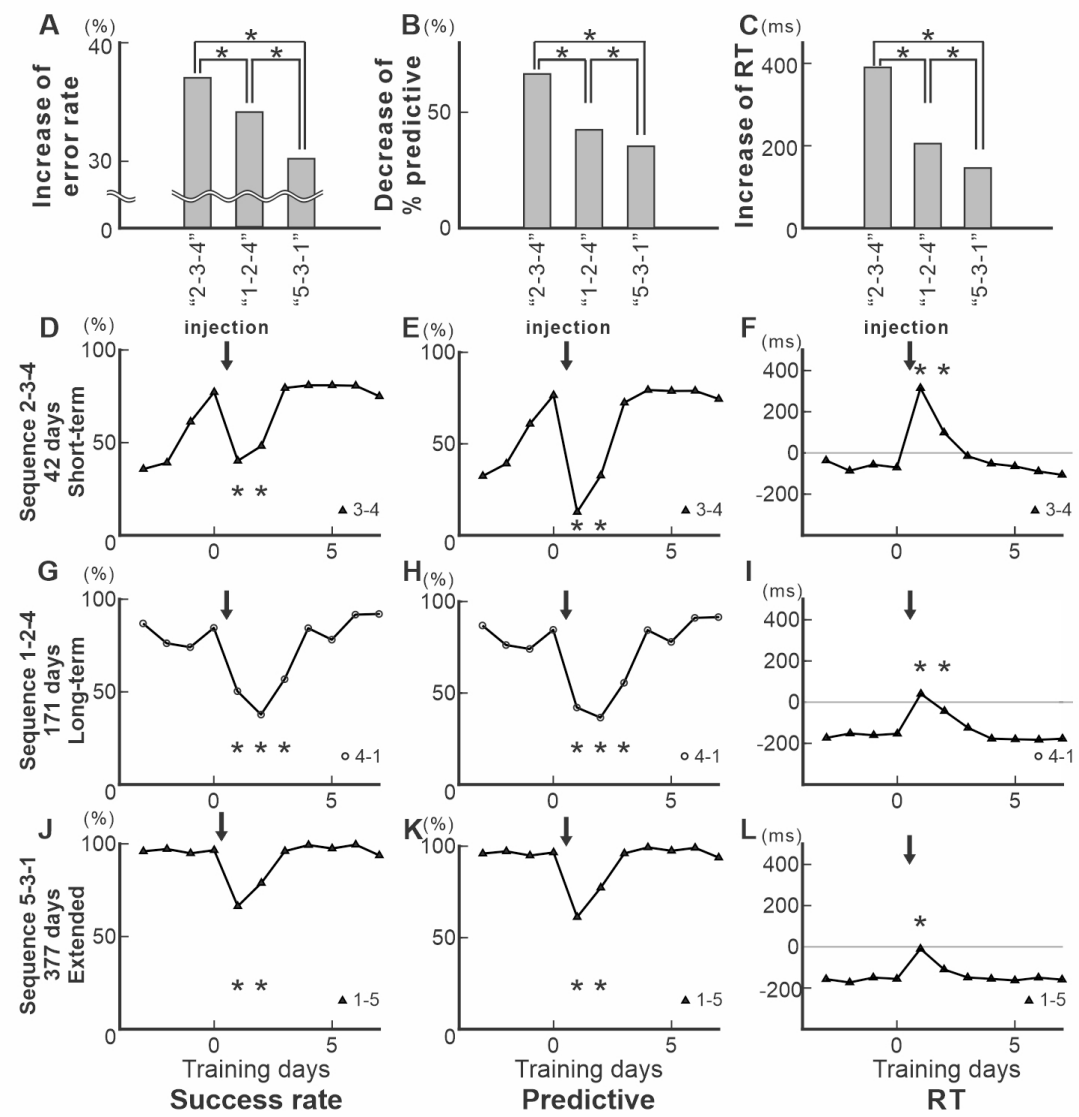
Performance in the Repeating task of the injection session shown in Fig. 3. A-C. Differences in performance measures of the most affected movement within each sequence, calculated as post-injection performance minus the pre-injection performance. A. Increase in error rates across the three sequences: ‘2–3-4’ (37.0%), ‘1–2-4’ (34.1%), and ‘5–3-1’ (30.2%). Significant differences were observed among all sequence pairs (generalized linear model [GLM] analysis, *p* < 0.001). B. Decrease in percentage of predictive trials across sequences: ‘2–3-4’ (63.9%), ‘1–2-4’ (42.4%), and ‘5–3-1’ (35.3%). Significant differences were observed among all sequence pairs (GLM analysis, *p* < 0.001). C. Increase in response times (RTs) across sequences: ‘2–3-4’ (385.6 ms), ‘1–2-4’ (193.4 ms), and ‘5–3-1’ (147.8 ms). Significant differences were observed among all sequence pairs (two-way ANOVA, *p* < 0.001). D–L. Time course of performance in the Repeating task from 4 days before to 7 days after anisomycin injection (arrows indicate injection). Anisomycin injection in M1 impaired task performance for 2–3 days post-injection, even after extended training. Performance recovered to baseline levels after 2–3 days of retraining. Asterisks denote significant differences from baseline (*χ*^*2*^ *test* for performance and predictive trials; *t-test* for RT; *p* < 0.05).

Notably, the effect of anisomycin injection on error rate was more pronounced for the ‘Short-term’ trained sequence compared to the ‘Long-term trained’ and ‘Extensively’ trained sequences (Fig. 4A; GLM analysis, *p*<0.001; see Methods). Similarly, the decrease in the percentage of predictive trials and the increase of the Response Time (RT) for the ‘Short-term’ trained sequence were significantly larger than those for the ‘Extensively trained’ sequence (Fig. 4B, C: GLM analysis for predictive trials, two-way ANOVA for RT, *p*<0.001; see Methods for the definition of predictive trials, RT and statistical analysis). These results suggest that the neural representation of ‘Long-term’ and ‘Extensively’ trained sequence is less susceptible to interference.

### Performance of Repeating sequences recovered after a few days of training.

After the injection, we continued daily training of the monkeys and monitored their performance. Figures 4D-L show performance data of the Repeating task from 4 days before to 7 days after the anisomycin injection session shown in Fig 3. Following the anisomycin injection into M1, the number of correct responses (Fig. 4D, G and J) and the percentage of predictive trials (Fig. 4 E, H and K) decreased across all three sequences of the Repeating task (*χ*^*2*^ *test, p* < 0.05). Additionally, the average Response Time (RT) increased for all three sequences (Fig. 4F, I and L) (*t-test*, *p*<0.05). However, after 2–3 days of training, the monkey’s task performance recovered to the baseline levels for all sequences of the Repeating task (Fig. 4) (*χ*^*2*^ *test* for performance and predictive, *t-test* for RT, *p*<0.05). No significant differences were observed in recovery periods between sequences with varying training durations. The performance recovery period following the protein synthesis inhibition was consistently short, 2–3 days (48–72 hours), regardless of training duration. This recovery timeline aligns with rodent studies showing that anisomycin injection inhibits protein synthesis in the injected brain area for 48 hours (40). These findings suggest that while protein synthesis inhibition disrupted memory traces around the injection site the effect on task performance is reversible with additional practice.

### The effect of protein synthesis inhibition is a specific consequence of the induced translational blockade during learning.

**Fig. 5.**
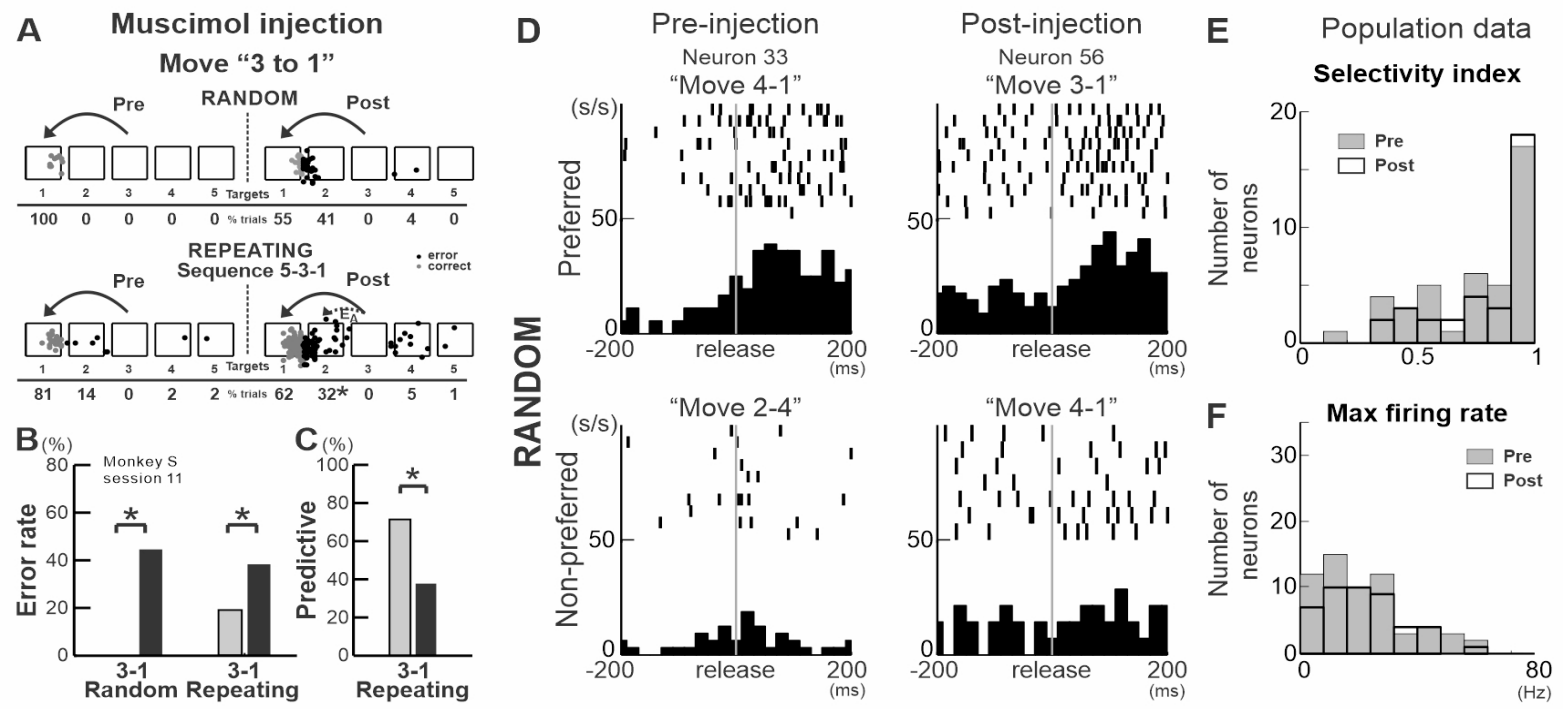
The observed behavioral effects (Fig. 3) were specifically due to protein synthesis inhibition affecting memory. A-C, The effect of muscimol injection in M1 on movements from target “3 to 1” of the Random and Repeating tasks. A. Left: pre-injection; Right: post-injection. gray: correct: black: error. B. Error rates. The number of error trials increased both in the Random and Repeating tasks after the injection (Random: pre 100%, post 55.4 %: *χ*^*2*^ *test, p* < 0.001; Repeating: pre 81.0%, post 61. 8%; *χ*^*2*^ *test, p* = 0.001). C. Number of predictive responses significantly decreased after the muscimol injection (pre 71.4%, post 37.8%; *χ*^*2*^ *test, p* < 0.001). D. Activity of two individual M1 neurons. Neural activity was aligned at the monkey’s release of the touch screen. Left: Activity of a neuron recorded before the anisomycin injection (neuron 33). Right: Activity of a neuron recorded 24 hours after the anisomycin injection (neuron 56). Upper panels: neural activity during the movements that had maximum firing rates (Neuron 33: during movement from target 4 to 1; Neuron 56: during movement from target 3 to 1). Lower panels: neural activity during the movements that had minimum firing rates (Neuron 33: during movement from target 2 to 4; Neuron 56: during movement from target 4 to 1). Both neurons were recorded 1 mm away from the injection site. Both neurons had movement selectivity in the Random task. E. Selectivity index of M1 neurons recorded before and after anisomycin injections (pre: n=62; post n=45). Selectivity Index, pre: mean=0.76, sd=0.24; post: mean=0.82, sd=0.22 (*t-test*, *p*=0.22, *df*=74). There was no significant difference in selectivity indexes between pre- and post- injection conditions. F. Maximum firing rate of M1 neurons recorded before and after anisomycin injections (pre: n=62; post: n=45). Maximum firing rate, pre: mean=21.73, sd=15.37, post: mean=21.59, sd=13.71 (*t-test*, *p*=0.96, *df*=104). Asterisks indicate *p* < 0.05.

Although anisomycin has been widely used in rodents to study memory systems, our studies are the first to apply it in non-human primates to investigate memory systems (45). We verified that the observed behavioral effects (Fig. 3) were specifically due to protein synthesis inhibition affecting memory, using three different approaches. First, we inactivated M1 by injecting muscimol, a GABA_A_ agonist, into the shoulder representation area of M1 and tested its effect on performance of the Random and Repeating tasks (Fig. 5A-C). The inactivation resulted in a significant increase in the number of incorrect responses both in the Random and Repeating tasks (Fig. 5B, C: *χ*^*2*^ *test, p*<0.01). The effect of injection was more pronounced for certain movements, and the same movements were affected in both the Random and Repeating tasks (increase of error rate: Random – move ‘3–1’: 44.6%, ‘5–3’: 1.8%, ‘1–5’: 17.6%; Repeating – ‘3–1’: 19.2%, ‘5–3’: 4.3%, ‘1–5’: −2.8%). In both tasks, the monkey’s reach fell short of the correct target after the muscimol injection (accuracy errors, 41% for Random; 32% for Repeating) (Fig. 5A, B). The increase in the number of direction errors after the injection was small in both tasks (Random, pre: 0%; post: 4%; Repeating, pre: 4%; post: 6%). Contrary to the effects of anisomycin, the effects of muscimol injections on task performance were primarily on the accuracy of movements in both the Random and the Repeating tasks (n=2 injection sessions in monkey S). The difference suggests that the deficit in task performance after the anisomycin injection is likely to be a specific consequence of the induced translational blockade, rather than any nonspecific inhibition or dysfunction of neural activity in M1.

Second, to validate the notion that the anisomycin effect is due to its inhibition of protein synthesis, we injected another type of protein synthesis inhibitor, cycloheximide (39). Anisomycin and cycloheximide have distinct mechanisms of action and different side effects (39). The injection of cycloheximide in M1 resulted in a significant increase in the number of incorrect responses during the Repeating task, but did not have an effect on performance of the Random task. The effect of injection was more pronounced for certain movements of Repeating sequences. During the Repeating task, the error rate increased from 3.6 % to 42.5% for the movement “1–2” for the sequence ‘1–2-4’ (*χ*^*2*^ *test, p*<0.05). On the other hand, the error rate of the movement ‘1–2’ performed during the Random task did not increase at all (error rate, pre: 0%, post: 0%). The consistency of the results with the two pharmacological agents suggests that the effect on behavior is due to the common effect of the agents, inhibition of protein synthesis.

Third, we recorded the activity of neurons before and after the anisomycin injections and evaluated the effect of injection on neural activity (Fig. 5D-F). Figure 5D shows activity of two M1 neurons during the Random task before and after injections. The neurons were recorded 1 mm away from the injection site. We analyzed neural data collected during the Radom task because the anisomycin injection caused a large increase in number of errors during the Repeating task, as we showed previously (Fig. 3), which prevented the fair comparison of neuron activity during similar movements. Both neurons had movement selectivity and similar maximum firing rates during the preferred movements. We recorded the activity of 107 M1 neurons before and after the injections (pre: n=62; post: n=45, recorded within 1–2 mm from the injection sites, pre: 7 sessions, post: 4 sessions) and obtained the maximum mean firing rate and a selectivity index (SI) for each neuron (Fig. 5E and F) (see Methods). There were no significant differences in these measures between pre and post injections (*t-test*, *p*>0.05) (Fig. 5E and F). The results suggest that the deficit in task performance after the anisomycin injection was likely to be a specific consequence of its translational blockade, rather than any nonspecific inhibition or dysfunction of neural activity.

These observations suggest that we successfully manipulated the sequence memory by injecting a protein synthesis inhibitor. The inhibition of protein synthesis in M1 disrupted the performance of memory guided sequential movements at all stages of learning without disrupting motor production. Observations from M1 inactivation using muscimol, recording of neural activity after the anisomycin injection and the injection of cycloheximide, another type of protein synthesis inhibitor, all support the conclusion that the deficit in the performance of the Repeating task after the anisomycin injection was a specific consequence of the induced translational blockade (43, 46), but not of nonspecific neural dysfunction.

### The effect of protein synthesis inhibition in M1 on memory guided sequential movements decreased as learning progressed.

**Fig. 6:**
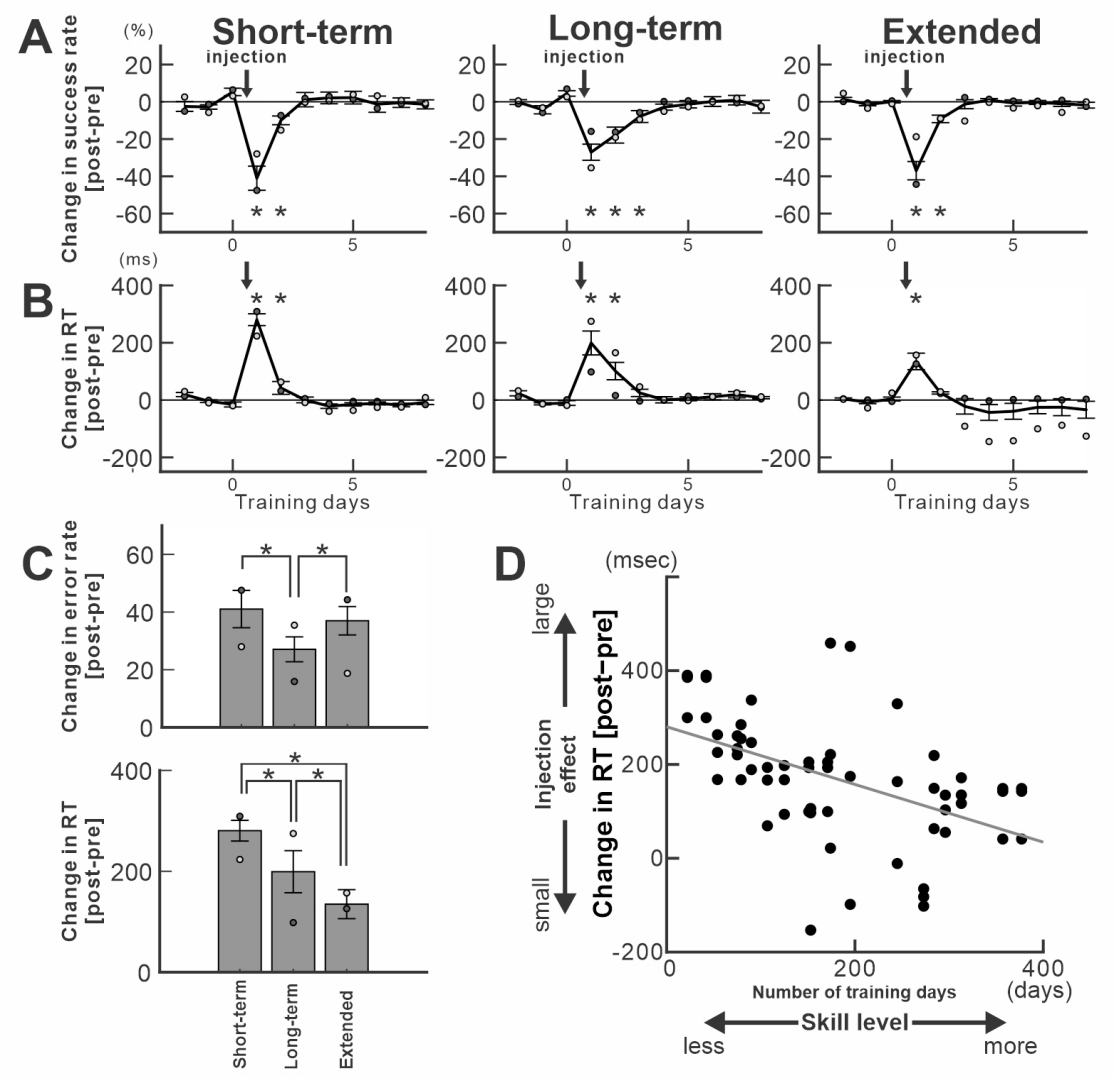
Effect of anisomycin injection on performance of the Repeating task (population data). A.B. Mean error rates (A) and mean response time (RT, B) of the most affected movements from 3 days before to 8 days after anisomycin injection (monkey N, n=5; monkey R, n=4). Arrows indicate injection timing. Light and dark gray dots denote the means for monkeys 1 and 2, respectively; error bars show se. Anisomycin injection in M1 affected both error rate and RT in the Repeating task for 2–3 days post-injection, even after ‘Extended’ training and performance returned to baseline after 2–3 days of training. C. Mean change in performance measures for Short-term, Long-term, and Extended trained sequences, calculated for the most affected movement within each sequence. Light and dark gray dots represent monkeys 1 and 2, respectively; error bars show s.e. Top: Increase in error rate: Short = 41.03%, Long=26.52%, Extended=36.98%. (*pairwise comparisons*: Short vs Long, *p* = 7.9 × 10⁻¹⁹, Short vs Extended, *p*=0.48, Long vs Extended, *p* = 6.1 × 10⁻¹⁹,). Bottom: Increase in RT: Short =280.47ms, Long=198.66ms, Extended=134.82ms. (*pairwise comparisons*: Short vs Long, *p* = 2.1 × 10⁻⁴; Short vs Extended, *p* = 7.2 × 10⁻⁴^*2*^; Long vs Extended, *p* = 2.9 × 10⁻^*25*^). D. Effect size of RT change was negatively correlated with training duration (*Pearson’s correlation coefficient, r*= - 0.4964, *p*=5.4791e-05). The x-axis represents the number of training days, while the y-axis indicates the effect size of RT change. Asterisks indicate *p* < 0.05.

The injection results in Figures 3 and 4 demonstrated that, within an injection session, the effect of protein synthesis inhibition in M1 varied with the training duration of each Repeating sequence. To determine whether this effect of protein synthesis inhibition in M1 is consistently correlated with the training duration, we repeated anisomycin injections after the monkey’s performance had recovered to the pre-injection level and tested its effect on performance of the tasks (Fig. 6, Monkey N, *n=5*; Monkey R, *n=4*). Injection sessions were separated by more than two weeks of training (see Methods), as performance recovered within 2–3 days of training regardless of training duration (Fig. 4). All injections were placed within the shoulder or elbow area of M1, but at slightly different sites to minimize the tissue damage. After each injection session, the monkeys were trained daily, and recovery of task performance was confirmed. The duration of recovery was consistently 2–3 days across all injection sessions and training durations: ‘Short-term’ (<100 days), ‘Long-term’ (100–200 days), and ‘Extended’ (>200 days) (Fig. 6A, B; see Methods). Overall, the effect of injections on the performance of the Repeating task was consistently observed and significant (*χ*^*2*^ test for error rate and predictive responses, *ttest* for RT, *p* < 0.05). In contrast, no significant decrease of performance was observed in the Random task (*p* > 0.05).

To examine whether the strength of the injection’s effect on performance depended on training duration, the most affected movement of each Repeating sequence from the two monkeys was classified into one of three groups based on training duration: ‘Short-term’ (< 100 days), “Long-term’ (100–200 days), and ‘Extended’ (> 200 days). Magnitude of changes in RT after the injection decreased as training proceeded (Fig. 6C). RTs were significantly different across all groups (see Methods for details; p<0.001 for all pairs) (Fig. 6C). These results indicate that the effect of injections on RTs depended strongly on training duration. On the other hand, changes in performance measured by success/error rates across training duration were not as consistent between two monkeys. Pairwise comparisons of error rates showed significant differences among all three groups for both animals (Monkey N, Short vs Long: *p*=1.678e-21; Short vs Extended: *p*=0.006036; Long vs Extended: *p*=1.756e-31; Monkey R, Short vs Long: *p*=1.942e-13; Short vs Extended: *p*=6.211e-26; Long vs Extended: *p*=1.348e-10; see Methods), but not always in the same direction (Fig. 6A). The inconsistent effects of training duration on error rates suggest that other regions involved in sequence learning, such as premotor areas, may compensate for M1’s disfunction differently across learning stages (5, 53, 67). For example, inactivation studies show that the pre-SMA contributes only to the very early stage of learning a new sequence structure (5, 53, 55). Other possible factors include uncontrolled elements of performance (e.g., motivation, the number of trials in a session, fatigue), the monkey’s option to avoid errors by waiting for the visual cue in the Repeating task, differences in reaching distances (e.g., long move ‘1–5’ vs short move ‘2–3’), variability in the likelihood of direction errors, or the non-linear improvement of success rates as movement chunking evolves across days. Consequently, changes in error rates are less robust, and persist for longer during training. Nevertheless, because protein synthesis inhibition disrupted success rate at all stages of sequence learning, our findings indicate that M1 contributes to learning of sequence structure throughout all stages of learning, potentially in collaboration with premotor areas in a stage-dependent manner.

We further examined whether the effect of injection on performance of the Repeating task is correlated with training duration, eliminating the use of arbitrary grouping based on training duration (See Methods). We found that the magnitude of the injection’s effect on the RT for each movement was significantly correlated with the training duration of a sequence (Fig. 6D; *Pearson’s correlation coefficient, r* = - 0.49644, *p* = 5.4791e-05). On the other hand, the magnitude of the injection’s effect on the error rate for each movement was not correlated with the training duration of a sequence (*Pearson’s correlation coefficient*, *r* = - 0.089275, *p* = 0.497). Furthermore, anisomycin injections had no significant effect on MT and performance of the Random task (i.e., visually guided reaching) (45). These findings strongly support M1’s role in speed improvement during extended repetitive practice and suggest that the transition time from one movement to the next becomes shorter, leading to shorter RT in our task.

The results show that the neural representations of ‘Long-term’ trained sequence and ‘Extended’ trained sequence are less susceptible to protein synthesis inhibition, and thus less vulnerable to interference in terms of speed. The decreased injection effects indicate that the rate of memory consolidation declines as learning proceeds. Our results suggest that the neural traces of sequential movements in M1 are gradually stabilized through repetitive practice to support a high level of performance.

### Consolidation during repetitive practice

**Fig. 7:**
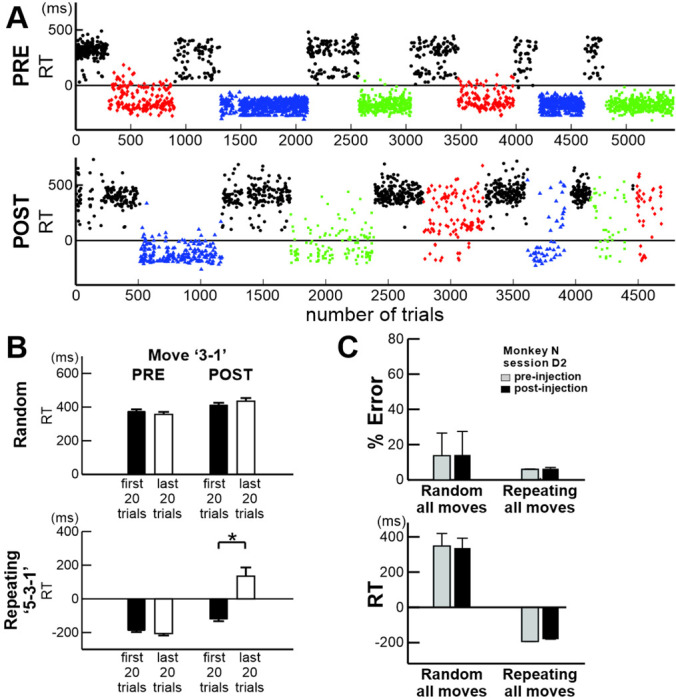
Effect of anisomycin injection on Response Time (RT). A. RTs in Random (black) and Repeating tasks (sequence ‘5–3-1’: blue triangles; ‘1–2-4’: green squares; ‘2–3-4’: red circles) before (top) and after (bottom) an anisomycin injection. RTs increased for all three sequences post-injection. B. Mean RTs for the first and last 20 trials of movement ‘3–1’ in Random (top) and Repeating (‘5–3-1’; bottom) tasks during pre- and post-injection sessions (*t-test*, early vs late trials: Random: pre *p*=0.55, post *p*=0.37; Repeating pre *p*=0.36, post *p*=0.002). C. Performance test 3 days after the anisomycin injection. There was no significant effect of injection on performance of the tasks and RTs. Top: Mean Error rate (*χ*^*2*^ test, *p*>0.05 for Random and Repeating). Bottom: Mean RT (*t-test*, *p*>0.05 for Random and Repeating). Error bars, s.e. Asterisks indicate *p*<0.05.

Analysis of Response Time (RT) provided further insight into this consolidation process during repetitive practice (Fig. 7; see Methods for RT definition). At the beginning of the post-injection session, RTs in both the Random and Repeating tasks were comparable to RTs observed in the pre-injection session (Random: ~360 msec; Repeating: ~ < 0 sec). However, as the session progressed, RTs increased only in the Repeating task. For example, in the Repeating sequence ‘5–3-1’ (blue), RTs increased incrementally across blocks, rising from negative values early in the session (mean of the first 20 trails of movement ‘3–1’: −118.50 ± 62.93 ms) to approximately 100–300 msec by the end (mean of last 20 trails: 135.00 ± 230.02 ms: *t-test* of first vs last, *p*<0.001; Fig. 7B). In contrast, RTs in the Random task showed no significant change within a session either before or after the injection (*t-test*, pre: *p*=0.55; post: *p*=0.37; Fig 7B). Likewise, RTs for the movement ‘3–1’in the Repeating task did not change significantly within a training session before the injection (*t-test*, *p*=0.30, Fig. 7B bottom). A significant increase in RT after the injection was observed for the affected movements of the Repeating task in eight of nine injections (*t-test*, *p*<0.05). These findings suggest that performing the task following the anisomycin injection disrupted the neural representation of the Repeating sequences in M1. This is consistent with findings of rodent studies proposing that synapses within the memory networks would be initially destabilized by protein degradation upon memory reactivation during task performance, and then restabilized through protein synthesis to update the memory network (35, 36, 41, 42, 44, 47–49).

We next asked whether exposure to anisomycin alone disrupts the performance of the Repeating task or whether performing the Repeating task within the drug effect window is necessary to disrupt performance of the Repeating task (Fig. 7C) (35, 36). We injected anisomycin into M1 of a highly trained monkey and assessed task performance after three days without training, at which time the injected anisomycin was no longer effective (40). We did not observe a significant decrease in task performance after three days of inactivity in either the Random or Repeating tasks (Fig. 8C; *χ*^*2*^ *test* for error rate and *t-test* for RT, *p* > 0.05, n = 2 injections). The results suggest that exposure of M1 to anisomycin alone did not disrupt the task performance but performing the Repeating task within the drug exposure window was necessary to disrupt the neural representations of sequential movements stored in M1. Our data suggest that anisomycin prevented the synthesis of the proteins needed to consolidate the neural representations of sequential movements (i.e., synaptic connections) in M1 during sequence practice as proposed in rodent studies (35, 36, 41, 42, 44, 47–49).

## Discussion

In this study, we trained monkeys to learn sequential movements and interfered with information storage in the primary motor cortex (M1) at multiple time points during learning by injecting a protein synthesis inhibitor, anisomycin, into the arm area of M1. The anisomycin injection disrupted the performance of memory guided sequential reaching at all stages of learning without affecting movement production. Notably, the effect of anisomycin injection on sequence performance was more pronounced for the ‘Short-term’ trained sequence compared to the ‘Long-term trained’ and ‘Extended’ trained sequences. Further analysis demonstrated that the magnitude of the injection effect on the RT of each movement was significantly correlated with the training duration of a sequence. These results suggest that the neural representation of ‘Long-term’ and ‘Extended’ trained sequence is less susceptible to protein synthesis inhibition and thus less vulnerable to interference. The decreased injection effects indicate that the rate of memory consolidation declines as learning proceeds and that the neural traces of sequential movements in M1 are gradually stabilized through repetitive practice to support a high level of performance.

Growing evidence demonstrates that M1’s activity changes with the learning of sequential movements both in the short term (within a training session to several months) (1–9, 12–15) and with years of training in humans and non-human primates (9, 11, 16–33). The changes in M1 after years of sequence training were reported in studies comparing musicians to non-musicians or comparing monkeys performing extensively trained sequential movements to those performing visually guided movements. However, it remains unclear from this work how learning-related plasticity occurs in M1, whether M1 is critically involved in sequence learning, and in which learning stage it may be. Changes of M1 activity after extensive training could be the outcome of plasticity during the early phase of the training. Our study using continuous long-term training and the staggered introduction of sequences covered learning stages within individual monkeys. Our results demonstrated that the protein synthesis inhibition disrupted the performance of memory-guided sequential reaching tasks at all stages of learning examined. This suggests that plasticity in M1 occurs at all stages of learning during repetitive practice over an extended period, continuously supporting learning of sequential movements.

The supplementary motor area (SMA) has long been considered responsible for the execution and maintenance of learned sequential movements (5, 9, 50–57). The SMA neurons exhibit sequence specific activity (52, 58–61). Inactivation or lesion of the SMA disrupted the performance of memorized sequential movements and inactivation or lesion of the pre-SMA disrupted the learning of a new sequence structure (53, 55, 62–66). In this view, M1 has been considered to relay the signal from the SMA to generate muscle commands for sequential movements. Additionally, inactivation of the dorsal premotor cortex (PMd) disrupted the performance of memorized sequential movements (67) and PMd neurons exhibit sequence preferential activity (10, 67). We cannot rule out the possibility that the SMA or PMd input to M1 (68–72) drives learning-related changes in M1. However, our results showed that protein synthesis inhibition disrupted the sequence performance at all stages of learning tested and suggest that aspects of learned sequences are consolidated within M1 at all times. M1’s contribution could be to enhance synaptic efficacy, which then leads to improving fluency of transitions between movements and the execution of individual movement components within a sequence. Further studies are needed to explore these possibilities.

Studies in rodents using protein synthesis inhibitors suggest that memory may be dynamically modified or reconsolidated upon retrieval (41, 42, 44, 73–76). Specifically, upon memory retrieval, synapses within the memory network are first destabilized by protein degradation during reactivation, followed by stabilization to update or strengthen the memory through protein synthesis (41, 42, 44, 75). Supporting this view, the inhibition of protein degradation by *clasto*-lactacystin-*β*-lactone (*β*lac) has been shown to prevent anisomycin-induced memory impairment (77). Moreover, human studies using TMS and functional imaging suggest that memory reconsolidation may occur in humans as well (78–80). Several factors, including the age and strength of a memory (47, 73, 81), the novelty of the experience (82–84), and duration of memory reactivation necessary for reconsolidation to occur (47, 48) are proposed as boundary conditions, which are considered to influence whether memory reconsolidation occurs.

In our study, we observed that the performance of the Repeating task was disrupted with a delayed onset of deficits after the anisomycin injection. The performance deficits may result from interference with the reconsolidation occurring during repetitive practice. The delayed onset of deficits (Fig. 7) may be linked to the duration of memory reactivation necessary for reconsolidation to occur (47, 48), specifically the duration for protein degradation to destabilize the memory network for reconsolidation. In fact, when we tested the injection effect outside of the drug’s effective window, three days after the injection without training, there was no significant effect on performance (Fig. 7C). The results suggest that protein synthesis inhibition alone may not be sufficient to disrupt memory-guided sequential movements. Consistent with rodent studies, memory reactivation during practice may be required to reveal the reconsolidation impairment due to protein synthesis inhibition.

Previous studies have shown that memory could be updated through reconsolidation when new information needs to be integrated (85–87), and that both the age and strength of a memory influence whether reconsolidation occurs or not (47, 48). Specifically, older and stronger memories (i.e., those reinforced through extended training) tend to be less sensitive to protein synthesis inhibition. In our study, the effect of protein synthesis inhibition on memory-guided sequence performance were smaller during the ‘Long-term and ‘Extended’ training phases in which RT variability was smaller (Fig. 1C). In our task, performance fluctuations due to movement variability may represent a form of “novelty” that serves as a boundary condition for reconsolidation. Our findings suggest that the memory network in M1 may be destabilized prior to update (or reconsolidation) at every practice rather than accumulating changes purely incrementally. This is consistent with the ‘lingering consolidation’ hypothesis, in which ‘the reactivation and reconsolidation cycle progressively stabilizes a memory’ (42). Furthermore, this would align with the gradual and continuous improvement of motor skill performance observed during extensive, repetitive practice. Recent studies in rodents have suggested that the motor cortex of rodents may not be essential for maintaining certain types of motor skills after “long-term” training (88–90). In contrast, our results demonstrate that protein synthesis inhibition in M1 of monkeys significantly impaired sequence performance even after more than 300 days of training (Figs. 3, 4 and 6). These differences may reflect several factors, including differences in whether the animals’ performance reached the boundary conditions of memory updating, the higher complexity of the sequential task used in our study, and species-specific anatomical differences (72). Further investigation will be needed to elucidate these possibilities.

Two-photon imaging studies offer insights into the possible mechanisms underlying memory-update in the motor cortex. In rodents, two photon imaging of the motor cortex during motor skill learning has shown the formation and growth of new dendritic spines and the elimination of old spines (91, 92). Additionally, two-photon imaging of cortical slices and electron microscopic studies revealed that protein synthesis is required for long-lasting synaptic plasticity (43) and spine-head enlargement and growth during learning (93, 94). The inhibition of protein synthesis resulted in a significant reduction in synapse number and synapse size in motor cortex of rodents in vivo (39). These observations suggest that, during motor skill training, unnecessary spines are eliminated through protein degradation, while the formation and growth of new spines are supported by protein synthesis which leads to enhanced neural efficacy. Thus, deficits in spine growth and formation may be associated with impairments in memory updates (i.e., consolidation), as observed in behavioral tests. Together with our findings, these results suggest that the neural traces for motor skills are continuously updated during repetitive practice through protein synthesis. The delayed onset of performance deficit, as shown in Figure 7, may reflect the time required for protein degradation to eliminate a sufficient number of spines to impact task performance (41, 42, 44).

Our study suggests that motor skill is gradually consolidated in M1 every time a subject practice and that the rate of consolidation (i.e., memory strengthening) becomes smaller as the learning proceeds. The lingering consolidation process implies that consolidation reaches a theoretical limit when the memory trace has become old and stable. Nevertheless, our results suggest that lingering consolidation still occurs in M1 in our monkeys after 377 sessions of practice on simple sequences. M1 may well make a steady contribution to the development of expertise, serving as the neural substrate underlying “Practice makes perfect”.

## Materials and Methods

### Behavioral task:

We trained three monkeys (*Cebus apella*, male or female, weighing 1~5 kg; > 2 years old) on the Random and Repeating tasks (Fig. 1) (32, 33, 45, 67, 95, 96). Each monkey was required to make sequential reaching movements to targets on a touch sensitive monitor with their right arms. In the Random task, the reaching movements were guided by visual targets displayed on a touch sensitive monitor in a pseudo-random order. Contact of the correct target triggered display of the next target after a 100 ms delay. After the monkey became proficient in performing the Random task (~50 days of practice), we introduced the Repeating task. In the Repeating task, new targets were presented according to a repeating sequence of 3 elements (e.g., 5–3-1–5-3–1- ..., Fig. 1A, B) 400 msec after contact of the preceding target. If the monkey made a correct response during the delay, the target is not shown and the task increments to the next target in the sequence. With practice, a monkey memorized and performed the sequence of the Repeating task using predictive responses instead of visual cues to direct their movements in more than 80% of trials. The two tasks were performed continuously in alternating blocks of 200–500 trials. The monkey received a liquid reward after every 4–5 correct responses. After the monkey became proficient in the performance of the first sequence (Fig. 1C), additional sequences (e.g., 1–2-4–1-2–4...) were introduced. The new sequences were introduced in a staggered design with 50–200 days intervals (Fig. 1D). This training design enabled us to compare the effect of pharmacological injection between the ‘Long-term’ trained and ‘Short-term’ trained sequences in a single injection session. Each monkey learned two to three sequences (Monkey N: ‘5–3-1’, ‘1–2-4’, ‘2–3-4’; Monkey R: ‘1–2-4’, ‘2–3-4’; Monkey S: ‘5–3-1’, ‘1–2-4’). When the monkey frequently stopped working during a training session, the session was omitted and the behavioral data were excluded from further analysis because an insufficient number of trials and conditions were collected. Control of the behavioral task and collection of movement data including videos of task performance were performed by PC computers running TEMPO software (Reflective Computing, Olympia, WA) and Matlab. Touch screen signals, task related events and neural activity were collected on-line at 1 kHz.

### Behavioral estimates of learning phases:

We used “Response Time” (RT) as the principal measure of sequence learning. We defined RT during the Random task as the time between the presentation of a new target and contact of that target. We defined RT during the Repeating task as the time between two targets touches minus the delay time, 400 msec (33, 45, 67, 96). We subtracted 400 ms to account for the delay in the cue presentation. This could result in a negative RT if the monkey moved quickly to the next target in the sequence before the presentation of a cue. RTs less than 150 ms were considered to be predictive. RTs less than 150 ms were chosen as a conservative cut-off for predictive responses as it is too fast for a simple reaction time to the visual cue. During the learning, an animal’s RT became shorter with practice (Fig. 7c, d). A negative RT indicates that the animal was internally generating the response before the visual cue was presented. After about 50 days of training on the Repeating sequence, the monkeys made predictive responses (RT < 150 ms) without using visual cues in more than 80% of the trials (Fig. 1C, D).

### Surgical Procedures:

All experimental procedures were conducted according to NIH guidelines and were approved by the IACUC of the University of Pittsburgh. We implanted a head restraint device, along with a MR compatible chamber for micro-injections and neural recording, on an animal’s skull using small screws and dental acrylic (45, 67). All surgical procedures were performed under general anesthesia using aseptic techniques. Anesthesia was induced with ketamine (10 mg/kg, IM) and maintained to surgical levels with 1–2.5% isoflurane. The animal received fluids throughout the surgical procedure (6–10 cc/hr, IV) and glycopyrolate (0.01 mg/kg, IM) to reduce secretions. Body temperature was maintained at 37–38 °C with a heating pad. We monitored body temperature, breathing, heart rate, end-tidal CO_2_, and O_2_ saturation. After the surgery, the animal received broad spectrum antibiotics (ceftriaxone, 75 mg/kg/day, IM, ~5 days) and analgesics (buprenorphine, 0.01 mg/kg, IM, ~3 days). The chamber’s placement over M1 was verified using structural MR images taken prior to and after the surgery (Fig. 2B). MR procedures for pretreatment, anesthesia, monitoring of vital signs and post-anesthesia monitoring were identical to surgical procedures, except that analgesic and antibiotic were not be given. After more than 2 weeks of recovery, we accustomed the animal to perform the tasks with its head restrained. When task performance returned to the pre-surgical level, we performed a craniotomy to expose the dura matter overlying M1 in the chamber using the same anesthetic techniques and safeguards used in the implant surgery. Animals were given dexamethasone (0.5 mg/kg divided into 3 doses, IM) on the day of the craniotomy surgery to prevent brain swelling.

### Intracortical stimulation:

We used intracortical microstimulation to identify the body part represented at each site in M1 and to physiologically define the border between M1 and the PMd (45, 67, 71). We used glass-coated micro-electrodes (0.6–2 MΩ at 1 kHz) to deliver intracortical microstimuli (45, 67, 71). A constant-current stimulator was used to deliver cathodal pulses (10–20 pulses, 0.2 ms duration, 333 Hz, 1– 40 μA) at a depth of 1500 μm below the cortical surface. Stimulus intensity was measured with a current monitor (Ion Physics). The motor response evoked by stimulation was determined by visual observation and muscle palpation. The response threshold was defined as the lowest stimulus intensity necessary to evoke a response on more than 50% of the trials (71, 97, 98). We systematically mapped M1 with micro-electrode penetrations spaced ~1.0 mm apart (except to avoid blood vessels) (Fig. 2).

### Micro-injections:

We made micro-injections of pharmacological agents (anisomycin, cycloheximide or muscimol) at selected sites in the forelimb area of M1 of a trained monkey (45, 67). We prepared solutions of anisomycin (100 μg/μl in ACSF, pH 7.2–7.4), cycloheximide (40 μg/μl in 20% ethanol/ACSF) and muscimol (5 mg/ml in saline) from commercially available powders (Sigma-Aldrich, MO). We injected the test solution at 1.5 mm below the cortical surface using a 30-gauge cannula connected to a 10 μl Hamilton syringe by applying slow pressure (e.g., over 10 minutes). Injection sites were placed more than 2 mm away from the border between M1 and PMd identified by microstimulation (Fig. 2). Previous studies using monkeys have shown that injection of 3 μl of muscimol into the cortex inhibited neural activity within a diameter of approximately 2–3 mm (66). Given that M1 in Cebus monkeys is more than 5 mm in width (Fig. 2), the effect of an injection of less than 3 μl of a pharmacological agent 2 mm away from the border was expected to remain confined within M1 (45). The cannula was left in place for more than 5 min to allow diffusion of the solution and prevent its reflux. For each anisomycin injection session, we injected a total of 5 μl of anisomycin solution into M1 of two monkeys. The solution was divided into two aliquots (2.6 μl and 2.4 μl) and injected at sites within M1 where microstimulation elicited elbow or shoulder movements. The injection volume was divided to minimize potential tissue damage and to ensure broader coverage of the cortical regions representing the elbow and shoulder. Task performance was assessed 20–24 hours after the injection and daily thereafter. Measurement of performance on days prior to an injection and/or on trial blocks preceding an injection was used as the baseline for comparison with post-injection performance. We repeated the anisomycin injections with behavioral testing five times in monkey N and four times in monkey R to test its effect on learning. To verify that the effect on behavior is due to the common effect of the agents (i.e., protein synthesis inhibition), we injected 3 μl of cycloheximide (40 μg/μl) at a site within M1 where microstimulation elicited elbow or shoulder movements. Cycloheximide blocks protein synthesis by a different mechanism than anisomycin. The effect of cycloheximide injection on tasks was tested 20–24 hours after the injection. Measurement of performance on a day prior to an injection and/or on trial blocks preceding an injection was used as the baseline for comparison with post-injection performance. Muscimol, a GABA_A_ agonist, (1–3 μl) was injected at a site within M1 of the monkey S where microstimulation elicited elbow or shoulder movements immediately after the collection of the baseline behavioral data and the effect of injection on task performance was tested 20 min after the injection to allow diffusion of the chemical in the brain tissue (*n*=2). After each injection session, the task performance was monitored every day until it recovered to the baseline to track the emergence of injection effects and to assess recovery from the drug’s effects. Injections were separated with more than 7 days and 5 training sessions. We examined whether the injection of the pharmacological agent impaired performance of the Random and Repeating tasks. During a post-injection test session, blocks of Random and Repeating trials alternated at frequent intervals to sample the animal’s performance evenly as the effect of the test substance emerged and intensified.

### Analysis of performance:

For every trial of each task session, we recorded various task parameters and measures of performance. The effect of an injection was assessed by examining the following: percentage of correct responses, types of incorrect responses, percentage of predictive responses (defined as RT < 150 msec), Response Time (RT) and Movement Time (MT) (45, 67). The errors recorded were: no hit, wrong target hit, background hit, or a corrective response (a correct response that immediately follows an error). MT is defined as the interval between the release of contact from one target to touch of the next target. We defined RT during the Random task as the time between the presentation of a new target and contact of that target. We defined RT during the Repeating task as the time between targets touches minus the delay time, 400 msec (see the section of Behavioral estimates of learning phases above)(33, 45, 67, 96). RT less than 150 ms was chosen as a cut-off for predictive responses as it is too fast for a simple reaction time to the visual cue. A wrong target hit during the Repeating task was categorized as two types: *accuracy errors* and *direction errors*. An *accuracy error* is defined as a reach performed in the correct direction, but to an endpoint outside of the correct target. This type of error suggests a deficit in motor production. A *direction error* was defined as a reach performed in the direction opposite to the correct target. This type of error suggests a deficit in selecting the movement component in the sequence. Direction errors could only occur for movements starting from targets on the center of the touch monitor (target 2, 3, 4), since movements in the opposite direction from targets at the edge of the touch monitor would fall outside the touch monitor. For statistical analysis, both types of errors were included in the calculation of performance error. An increase in RT and decrease in the number of predictive responses suggest an increase in the time for movement selection. Corrective responses were removed from analysis because the target is predictable after an error. We also monitored movement kinematics during the task using high speed video recording (100 Hz, Basler Inc., PA) in the frontal plane. For statistical analysis, we used *χ*^*2*^ tests with Holm–Bonferroni’s correction to examine the significance of changes in success rate and predictive responses in a session. We used *t-tests* with Holm–Bonferroni’s correction to examine MT and RT changes. To test the reproducibility of the task performance results, we repeated the injections five times in monkey N and four times in monkey R. In monkey R, we performed three additional injections to examine the effect of anisomycin injection on neural activity (see the Electrophysiology section of the Methods). To compare the strength of the injection effect between the sequences in the different learning phases, we fitted a generalized linear model (GLM), using either performance or predictive responses as the variable and the learning phase as a predictor for analysis shown in Fig. 4A and B. A binomial distribution with a logit link function was applied. For analysis in Fig. 4C, we used two-way ANOVA with RT and the learning phases as factors. To compare the strength of the injection effect between the sequences in the different learning phases as a population, the most affected movement of each Repeating sequence from two monkeys was classified into one of three groups based on the training duration: Short-term (less than 100 days, large RT decrease and large RT variability), Long-term (100– 200 days, smaller RT decrease and large RT variability), Extended (more than 200 days, small RT decrease and small RT variability) (Fig. 6A-C). These groupings were defined according to the learning curves shown in Fig. 1. In Fig 6C, we compared changes in performance measures of the most affected movements of each sequence across the Short, Long, and Extended groups using pairwise statistical tests (5 injections for monkey N and 4 injections for monkey R). RT was analyzed using a linear mixed-effects models with Condition (pre vs post) and Group (Short, Long, Extended) as fixed effects and InjectionID as a random effect to account for repeated measurements within each injection session. Pairwise comparisons of the Condition effect (post-pre) between groups were performed by testing linear contrasts on the interaction coefficients. Performance (correct/error) was analyzed using a logistic generalized linear mixed-effects model (logit GLME) with Condition (pre vs post) and Group (Short, Long, Extended) as fixed effects and InjectionID as a random effect. Pairwise comparisons for the Condition effect between groups were performed same as the RT analysis. For the *Pearson’s correlation coefficient* analysis between the training duration and the effect of the injection on RT (Fig. 6D), data from all the movements performed in the Repeating sequences were included. For the RT analysis examining changes within a training session (Fig. 7A, B), the first 20 trials and the last 20 trials of each movement in each session were compared using a *t-test*. Matlab (Mathworks) was used for analysis.

### Electrophysiological recording and analysis of neural activity:

To evaluate the effect of pharmacological agents on neural activity, we recorded extracellular activity of single neurons in M1 using glass-coated Elgiloy electrodes (0.6–1.5 MΩ). We recorded neural activity before and after the injection of anisomycin while the monkey S performed the tasks. As shown in Fig. 3, an anisomycin injection disrupted performance of the Repeating task on up to 80% of trials. Therefore, to evaluate the effect of injection on neural activity, we limited analysis on the data collected during the Random task. Neural data after the anisomycin injection were obtained during four injection sessions. Of these, performance data of three injection sessions were excluded from behavioral analysis because of insufficient number of trials in the Repeating task. The sample of neurons was taken 20–24 hours after the injection within 1–2 mm of the injection site from regions of M1 where intracortical stimulation evoked shoulder, elbow or wrist movements at thresholds lower than 25 μA. For each neuron, we used maximum firing rate and a selectivity index (SI) to assess the effect of injections. For each Random move, we measured the mean firing rate of a neuron in a 200 msec interval centered on target contact or release. We used this value to calculate a SI for each neuron: SI = [Max - Min]/ [Max + Min]. For statistical analysis, we used *t-test* to examine changes in SI and max firing rate between the group of M1 neurons recorded before and after the injections.

## Data Availability

All data needed to evaluate the conclusions in the paper are present in the paper. The data in other format can be provided by M.O. pending scientific review and a completed material transfer agreement. Requests for the data should be submitted to: machiko@pitt.edu.
